# The relationship between the use of fillers and Japanese proficiency: Focusing on OPI level assessment

**DOI:** 10.1371/journal.pone.0360747

**Published:** 2026-10-16

**Authors:** Zuohao Chen

**Affiliations:** Graduate School of Humanities, The University of Osaka, Osaka, Japan; King Mongkut’s University of Technology North Bangkok, THAILAND

## Abstract

This study employed a Cumulative Link Mixed Model (CLMM) to examine the relationship between Japanese learners’ filler use and OPI (Oral Proficiency Interview) level ratings. The results showed no significant association with proficiency for the *i*(い)-group, *u*(う)-group, *ano*(あの)-group, and *eeto*(ええと)-group fillers. In contrast, the *a*(あ)-group, *e*(え)-group, *o*(お)-group, and *n*(ん)-group fillers were significantly negatively associated with higher proficiency, while the use of *maa*(まあ)-group and *sono*(その)-group fillers was significantly positively associated with higher proficiency. Furthermore, an analysis of *maa*(まあ)-group and *sono*(その)-group fillers based on their functions and discourse roles showed that turn-internal *maa*(まあ) and inferential/re-presenting *sono*(その) require at least two clauses to be produced. This indicates a connection with paragraph-level language production, which is one of the criteria for ‘Text Type’ in OPI assessment. It was also found that the use of these fillers with such functions is significantly positively associated with higher proficiency. On the other hand, for fillers such as *ano*(あの) and *eeto*(ええと), as well as *maa*(まあ) used in adjacency pairs, no significant increase with proficiency was observed, confirming that their use is not associated with paragraph-level production. The use of fillers linked to paragraph-level production can be considered one of the key linguistic factors that can help explain the relationship between filler use and the development of oral language proficiency.

## 1. Introduction

Japanese fillers such as *eeto*(ええと) and *ano*(あの) have also been called ‘hesitation markers’ or ‘place-holding words,’ and have traditionally been regarded as meaningless expressions that fill the gaps between utterances. In recent years, however, their functional roles have become clearer, and fillers are now considered essential elements for carrying out real-time spoken communication. It has been argued that they need to be actively taught to learners [1]. Although it is thought that more proficient learners can use fillers more appropriately, it remains unclear what linguistic factors can explain the relationship between filler use and the development of oral language proficiency. Therefore, to clarify this issue, the present study analyzes learners’ utterance data by proficiency level, taking into account the usages and functions of fillers.

## 2. Oral proficiency and its assessment

Proficiency indicates the degree of acquired knowledge or skill and is used in second language education to evaluate learners’ abilities.

Frameworks for assessing Japanese proficiency include the JLPT and the JF Standard for Japanese-Language Education (based on the CEFR). In addition, the OPI (Oral Proficiency Interview), specifically targets oral ability [2]. Developed by ACTFL, the OPI is a cross-linguistic interview-style oral test. It sets levels of Novice, Intermediate, Advanced, and Superior, with Novice through Advanced further divided into Low, Mid, and High. Testers present topics of varying difficulty, and the examinee’s level is determined from their language output [3, pp. 66–68]. The test demonstrates the relationship between proficiency and the range of topics a learner can handle, as summarized by Yamauchi [3, pp. 23–24].

Novice: Basic everyday matters
Intermediate: Predictable topics directly related to daily life
Advanced: Topics beyond one’s immediate living sphere, explaining personal experiences and interests
Superior: Topics of broad general interest or specific areas of interest/expertise

Furthermore, Yamauchi [3,p. 40] summarizes the range of language that each OPI proficiency level can control as follows.

Novice: Cannot produce sentences stably
Intermediate: Can produce sentences stably
Advanced: Can produce paragraphs stably
Superior: Can produce multiple paragraphs stably

Given the above, it is understood that while advanced learners at the OPI level can produce language at the paragraph level even on relatively complex topics, novice learners struggle to produce sentences stably even on relatively simple topics.

Previous research based on OPI interview data has demonstrated a clear relationship between filler use and proficiency [4]; see the Previous Research on Learner Filler Use section]. To clarify the relationship between filler use and Japanese oral proficiency, it is important to analyze filler use based on the criteria used to determine proficiency levels—i.e., the test’s rating criteria. Makino [5,p. 11] presents the rating criteria for OPI proficiency levels as shown in Table 1 (translated by the author).

**Table 1:** OPI Level Rating Criteria [5,p. 11].

Level	Functions / Global Tasks	Contexts / Topics	Text Type
Superior	Can speak both concretely and abstractly, provide supported opinions, formulate hypotheses, and handle unfamiliar situations.	Formal and informal contexts; topics of broad general interest, special areas of interest, and specialized topics.	Multiple paragraphs
Advanced	Can provide detailed descriptions and narrations; handle unexpected situations or negotiations.	Most informal contexts and some formal contexts; personal and general topics.	Paragraph
Intermediate	Can say and respond to what one intends to communicate; handle routine situations and transactions.	Some informal contexts; predictable, familiar topics directly related to one’s immediate surroundings.	Sentence
Novice	Can communicate minimally using formulaic expressions, memorized phrases, and lists.	Routine informal situations; very common everyday content.	Word, chunks

According to Table 1, the main categories of OPI level rating criteria include ‘Functions/Global Tasks,’ ‘Contexts/Topics,’ and ‘Text Type.’ Focusing on ‘Functions/Global Tasks’ and ‘Contexts/Topics,’ it can be observed that proficiency increases as learners become able to handle more complex topics and contexts. Makino [5,p. 11] states that learners’ language proficiency in the topics and contexts discussed above ultimately manifests in the concrete form of ‘Text Type.’ The ‘Text Type’ category is further subdivided into four levels — ‘Word/chunks,’ ‘Sentence,’ ‘Paragraph,’ and ‘Multiple paragraphs’ — which correspond to the OPI levels of Novice, Intermediate, Advanced, and Superior, respectively. Among these, ‘Paragraph’ is defined as a collection of sentences cohesively connected [5]. Yamauchi [3,p. 40] notes that ‘Text Type’ refers to the kind of discourse a learner can produce. This ‘Text Type’ represents the range of language that learners can control and serves as the foundation of the OPI’s view of language acquisition [3,p. 40].

Meanwhile, fillers are also known to have ‘functions related to text construction,’ such as marking boundaries between sentences [6,p. 40; see the Previous Research on Filler Usages and Functions section]. Learners’ oral proficiency is also closely related to text construction ability. By clarifying the relationship between the use of fillers and language proficiency, it is expected that in the teaching of spoken language, fillers will be more actively taught as a linguistic resource that supports the stable production of sentences and paragraphs, and that learners will use fillers more consciously and effectively.

## 3. Previous research and position of the present study

### 3.1. Previous research on learner filler use

Japanese speakers frequently use fillers such as *ano*(あの), *eeto*(ええと), and *maa*(まあ) in conversation. The use of these fillers is thought to reflect the speaker’s ongoing cognitive activity [7]. Fillers can also serve communicative functions; for example, in making requests, verbalizing the cognitive activity of ‘considering how to say something’ as *ano*(あの) can increase politeness [8,pp. 42–43]).

Because filler use reflects cognitive activity and serves communicative functions, it has been examined in Japanese language education as a window into learners’ language acquisition processes. These studies suggest that filler use is closely associated with language proficiency.

For instance, Yamauchi [4], after observing filler use across proficiency levels, supports the view that learners who can appropriately use *maa*(まあ) are at the ‘Advanced-Mid’ level on the OPI. Yamauchi [4,p. 42–45] also argues that fillers should be addressed in language education, identifying *ano*(あの) and *eeto*(ええと) as items to be acquired at the novice level, and *maa*(まあ) and *sono*(その) as items to be introduced from the intermediate level onward.

Konishi [9] reports that as proficiency increases, some fillers increase in frequency while others decrease. This study groupified fillers with lexical content such as *ano*(あの), *eeto*(ええと), and *maa*(まあ) as ‘lexical fillers,’ and vowel-based fillers such as *a*(あ), *i(*い), *u*(う), *e*(え), *o*(お), and *n*(ん) as ‘vowel fillers,’ analyzing learner use in spoken discourse. The results showed that as proficiency increases, the use of vowel fillers decreases while the use of lexical fillers increases.

Analysis of filler use thus holds significant implications for understanding learners’ language acquisition processes and assessing communicative competence. However, previous research has not yet sufficiently clarified what linguistic factors may help explain the relationship between filler use and the development of oral language proficiency—for example, why learners who appropriately use *maa*(まあ) tend to be rated at the ‘Advanced-Mid’ level (whereas other fillers do not show this pattern), and what constitutes appropriate use of *maa*(まあ).

### 3.2. Previous Research on Filler Usages and Functions

The Oral Proficiency and Its Assessment section showed that ‘Text Type’ underlies the OPI’s view of language acquisition. Meanwhile, fillers have also been noted to possess ‘functions related to text construction,’ such as marking boundaries between sentences [6,p. 234]. This suggests that fillers are an important linguistic resource for learners in modulating and controlling language production at the ‘sentence’ and ‘paragraph’ levels. Therefore, the uses and functions of fillers should also be considered when examining the relationship between filler use and Japanese oral proficiency. This section reviews these usages and functions to provide clues for the analysis.

Regarding vowel fillers such as *a*(あ), *i*(い), *u*(う), *e*(え), *o*(お), and *n*(ん), Yamane [6,p. 237] notes that their primary function is to adjust utterance rhythm and fill pauses, while their overuse can hinder listenability [6,p. 234]. Ishiguro [10] points out that the use of these fillers is influenced by learners’ native language and can be a source of unnaturalness.

Previous studies provide insights into how the occurrence of fillers such as *ano*(あの), *sono*(その), *eeto*(ええと), and *maa*(まあ) relates to preceding and following context. These studies include Daikuhara [1], Koide [11], and Takagi & Morita [12].

Daikuhara [1] analyzes *ano*(あの) and *sono*(その) from the perspective of continuity with demonstratives, comparing their usages. For *ano*(あの), Daikuhara [1] assumes it is linked to the cognitive activity of ‘having a message to utter and being in the process of formulating it,’ and that it searches for information within the ‘direct experience domain’ (where perceptual information and event memories reside). Analysis of constructed examples revealed that *ano*(あの) does not show strong continuity with the *a*(あ)-series demonstratives and has no special constraints on its environment. For *sono*(その), assuming it searches for information within the ‘indirect experience domain’ (where information obtained from context or inference is stored), Daikuhara [1] identified three usages: re-presenting type, inferential type, and indefinite type. Among these, the re-presenting type reintroduces a previously mentioned item or its equivalent; the inferential type introduces new information through inference; and the indefinite type covers cases not explained by the other two.

Koide [11] states that *eeto*(ええと) is involved in discourse management. It indicates that the speaker has noticed that the necessary information or linguistic form has not yet been formulated, signals entry into a mental activity to fill that gap, and functions to clear the previous mental content.

Takagi & Morita [12] analyze the functions of *maa*(まあ) based on its position of occurrence. Results show that *maa*(まあ) used at turn-initial positions in the first part, second part, or after the second part of an adjacency pair is produced when the speaker identifies an issue in the prior context or immediate situation and addresses it. *Maa*(まあ) occurring turn-internally is produced when the speaker identifies an issue with their own immediately preceding utterance and addresses it, or when an utterance deviates from the speaker’s expectations.

A review of previous research on filler usages and functions shows that fillers are an important linguistic resource for learners in modulating and controlling language production at the ‘sentence’ and ‘paragraph’ levels. As such, filler usages and functions are considered crucial clues for examining the research issues presented in the Previous Research on Learner Filler Use section.

### 3.3. Position of the present study

The Previous Research on Learner Filler Use section showed that previous research on filler use has not yet sufficiently clarified what linguistic factors may help explain the relationship between filler use and the development of oral language proficiency.

The Previous Research on Filler Usages and Functions section made two key points. First, while ‘Text Type’ underlies the OPI’s view of language acquisition, fillers also function to mark boundaries between sentences. Second, filler usages and functions provide crucial clues for examining the research issues presented in the Previous Research on Learner Filler Use section. Based on the above, the relationship among filler use, their usages/functions, and oral proficiency assessment is shown in Fig 1.

**Fig 1:**
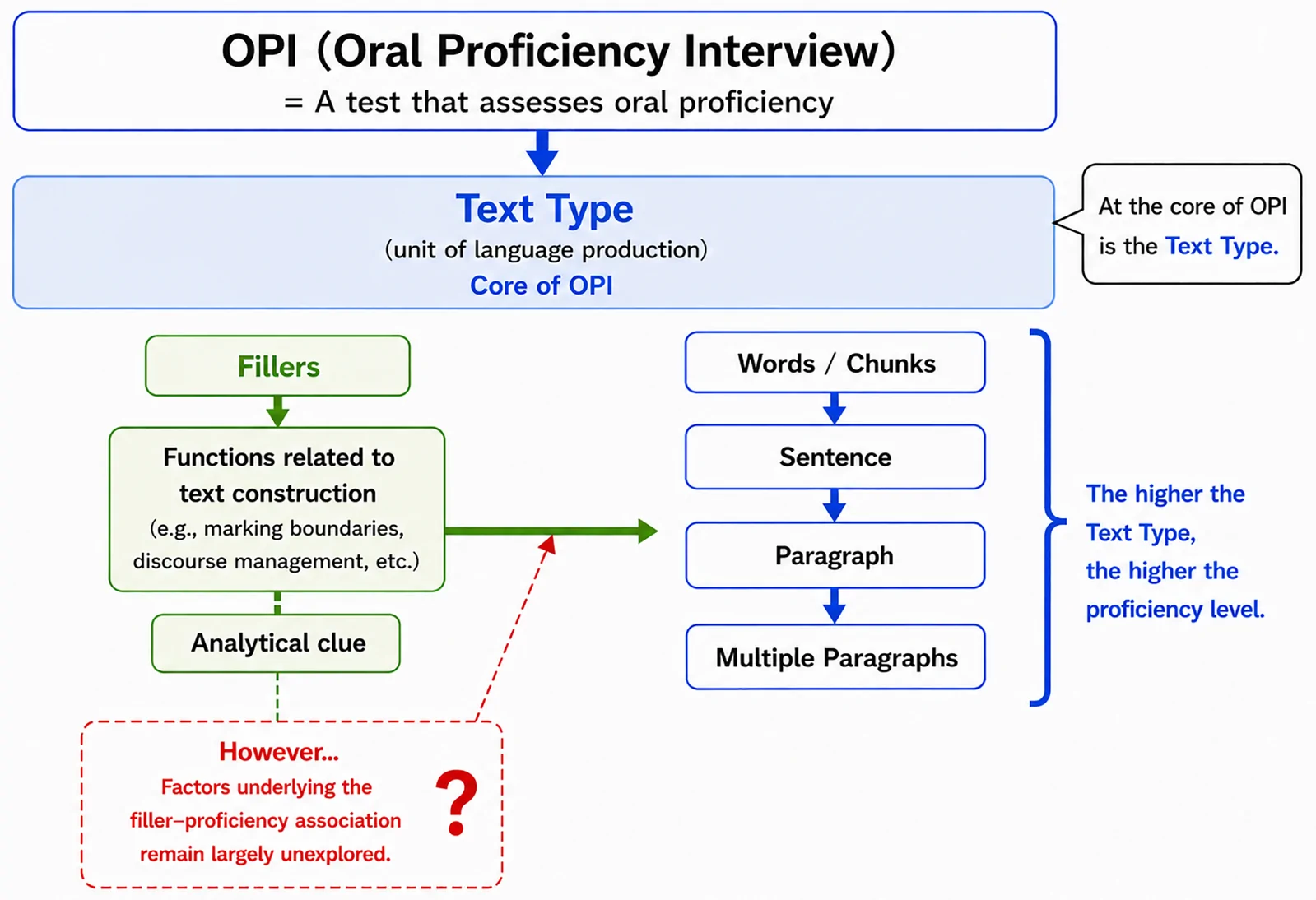
Conceptual Framework of the Relationship Between Filler Use and Oral Proficiency.

Previous studies have revealed that filler use is associated with learner proficiency and have identified the discourse functions of individual fillers. However, the linguistic factors underlying the relationship between filler use and oral proficiency remain largely unexplored. More specifically, while OPI research has established that ‘Text Type’ underlies proficiency assessment, the relationship between filler use and ‘Text Type’ has not been sufficiently examined. The novelty of this study lies in integrating filler research and OPI research—which have largely been treated independently—by analyzing filler use from the perspective of ‘Text Type,’ an OPI rating criterion. This approach offers a new perspective on the linguistic factors that can help explain the relationship between filler use and the development of oral language proficiency.

Based on the above, this study aims to clarify what linguistic factors can help explain the relationship between filler use and the development of oral language proficiency. The analysis will be conducted with reference to ‘Text Type’ and filler usages and functions.

## 4. Methods

### 4.1. Participants

This study used the ‘Japanese Learner Conversation Database’ [13] of the National Institute for Japanese Language and Linguistics, recorded within the ACTFL-OPI framework. The analysis targets 339 learners whose utterance data are publicly available. Learners’ native languages include Chinese, Korean, English, among others, totaling 42 languages. The proficiency level breakdown is as follows: Novice: 32 (High: 21, Mid: 10, Low: 1); Intermediate: 188 (High: 68, Mid: 84, Low: 36); Advanced: 110 (High: 24, Mid: 34, Low: 52); Superior: 9. Morphological analysis was conducted primarily using the modern spoken Japanese corpus Unidic [14]. The analysis yielded a total of 823,187 words (excluding punctuation). For the fillers *ano*(あの), *sono*(その), and *maa*(まあ), since they were occasionally misanalyzed as adnominal adjectives (the former two) or as an adverb (*maa*(まあ)), the counts for these fillers were manually verified in all cases.

### 4.2. Fillers analyzed

The selection of fillers analyzed in the present study was based on established classifications and previous empirical studies of Japanese fillers, particularly Yamane [6] and Konishi [9]. Konishi [9], drawing on Yamane [6]’s classification, classifies the fillers analyzed in learner speech into two types: lexical fillers (e.g., *ano*(あの), *eeto*(ええと), and *maa*(まあ)) and vowel fillers (e.g., *a*(あ), *i(*い), *u*(う), *e*(え), *o*(お), and *n*(ん)). The present study adopts these classifications and analytical decisions as the basic framework for selecting the fillers to be analyzed.

However, Konishi [9] does not include *sono* (その), whereas Yamauchi [4, pp. 49–51] lists *sono* (その) as a filler that should be introduced from the intermediate level onward. Given its relevance to the relationship between filler use and proficiency, the present study therefore includes *sono* (その) as an additional item for analysis.

In addition, in analyzing learners’ actual use of fillers, Konishi [9], while referring to Yamane [6] ‘s classification, treats *hai*(はい) and *un*(うん) as response tokens and excludes them from analysis. This study follows this approach and excludes *hai*(はい) and *un*(うん) as response tokens.

Furthermore, observation of actual conversational data based on the fillers discussed in Yamane [6,pp. 49–51] also revealed that some fillers, such as *moo*(もー) and *kono*(この), did not occur with sufficient frequency for analysis; these were also excluded.

Based on the above criteria, Table 2 lists the fillers analyzed in this study.

**Table 2:** Fillers Analyzed.

group	Orthographic Forms	Frequency	group	Orthographic Forms	Frequency
a(あ)-group	a(あ),aa(あー)	23,659	n(ん)-group	n(ん),nn(んー)	18,292
i(い)-group	i(い),ii(いー)	787	ano(あの)-group	ano(あの),anoo(あのー)	11,289
u(う)-group	u(う),uu(うー)	717	sono(その)-group	sono(その),sonoo(そのー)	1,471
e(え)-group	e(え),ee(えー)	5,796	eeto(ええと)-group	eeto(ええと),eto(えと)	4,552
o(お)-group	o(お),oo(おー)	1,268	maa(まあ)-group	ma(ま)、maa(まあ)	5,004

### 4.3. Analytical procedure

This section describes the analytical procedure. To address the research question, the relationship between filler use and proficiency must first be examined. However, in examining this relationship, two issues must be considered: (1) the intervals between proficiency levels are not necessarily equal [15], and (2) filler use may be influenced by learners’ L1 [9].

One analytical approach that addresses these issues is the Cumulative Link Mixed Model (CLMM), which assumes a continuous latent variable underlying the ordinal scale (i.e., proficiency levels) and incorporates random effects [16]. In this study, learners’ L1 is included as a random effect to account for L1-related differences.

Based on the above, the CLMM Analysis section first examines the relationship between filler use and proficiency using CLMM. The Discussion of the CLMM Results section discusses the results of the CLMM analysis. The Discussion Based on Filler Usages and Functions section then discusses the results from the perspective of filler usages and functions. Finally, the Conclusion section summarizes the findings.

## 5. CLMM analysis

### 5.1. Model specification for the CLMM

The CLMM analysis was performed using the *ordinal* package in R [17]. For the analysis, the frequency of each filler from the 339 learners was converted to an adjusted frequency per 1,000 words and entered as predictor variables. The OPI level (ordinal categorical data) was used as the response variable, and learners’ L1 was set as a random intercept. For the analysis, OPI levels from Novice to Superior were converted to numeric values from 1 to 10; this variable is denoted as ‘opi.’ The R formula is shown below, and the correspondence between OPI levels and opi scores is presented in Table 3.

**Table 3:** Correspondence Between OPI Levels and opi Scores.

opi Score	OPI Level	opi Score	OPI Level
10	Superior	5	Intermediate-Mid
9	Advanced-High	4	Intermediate-Low
8	Advanced-Mid	3	Novice-High
7	Advanced-Low	2	Novice-Mid
6	Intermediate-High	1	Novice-Low

r opi_ordered ~ ano + a + i + u + eeto + e + o + n + maa + sono + (1 | L1)

### 5.2. Results of the CLMM analysis

The Threshold Coefficients in the CLMM serve as an index for identifying the location of boundaries between proficiency levels, and are an important indicator for interpreting the relationship between filler use and proficiency. The results are shown in Table 4.

**Table 4:** Threshold Coefficients for OPI Levels.

	Increment	Estimate	Std. Error	z value	95% CI
1|2	–	−10.374	1.117	−9.288	[-12.564,-8.186]
2|3	+2.559	−7.815	0.569	−13.728	[-8.930,-6.699]
3|4	+1.641	−6.174	0.453	−13.614	[-7.062,-5.285]
4|5	+1.467	−4.707	0.379	−12.421	[-5.449,-3.964]
5|6	+2.082	−2.625	0.301	−8.727	[-3.215,-2.036]
6|7	+1.403	−1.222	0.274	−4.452	[-1.760,-0.684]
7|8	+1.185	−0.037	0.274	−0.135	[-0.573,0.499]
8|9	+1.126	1.089	0.302	3.609	[0.498,1.681]
9|10	+1.618	2.707	0.420	6.450	[1.885,3.530]

In Table 4, the vertical bar ‘|’ on the left side indicates the boundary between proficiency levels; for example, ‘1|2’ represents the boundary between OPI scores 1 and 2. The Estimate column shows the estimated value of each threshold (boundary location), and Increment shows the difference between adjacent thresholds. The estimated thresholds increased monotonically from −10.374 to 2.707 across the ten OPI categories.

Table 5 presents the estimation results of the CLMM (full model). The full model, which included all fillers as fixed effects, had a log-likelihood of −536.824 (df = 20) and an AIC of 1113.648. The null model, which included no fixed effects, had a log-likelihood of −676.000 (df = 10) and an AIC of 1372.200. The likelihood ratio test showed a statistically significant difference in model fit between the full and null models (LR χ² = 278.52, p < 0.001), indicating that including fillers as fixed effects improved model fit.

**Table 5:** Estimates for Each Filler in the CLMM.

	Estimate	Std. Error	z-value	p-value	VIF	95% CI
*a*(あ)-group	-0.033	0.003	-9.100	<0.001	1.259	[-0.040,-0.026]
*i*(い)-group	-0.051	0.106	-0.481	0.630	1.082	[-0.259,0.157]
*u*(う)-group	0.067	0.073	0.924	0.356	1.082	[-0.075,0.209]
*e*(え)-group	-0.028	0.009	-3.125	0.002	1.184	[-0.045,-0.010]
*o*(お)-group	-0.147	0.048	-3.040	0.002	1.220	[-0.242,-0.052]
*n*(ん)-group	-0.049	0.006	-7.808	<0.001	1.129	[-0.061,-0.037]
*sono*(その)-group	0.226	0.055	4.128	<0.001	1.111	[0.119,0.334]
*maa*(まあ)-group	0.028	0.013	2.092	0.036	1.151	[0.002,0.054]
*eeto*(ええと)*-*group	0.003	0.008	0.444	0.657	1.130	[-0.012,0.018]
*ano*(あの)-group	0.006	0.004	1.308	0.191	1.079	[-0.003,0.015]
AIC:1113.648 log Lik.:-536.82(df=20)						
Variance: <0.001 Std.Dev.:<0.001						

Next, a minimal model was constructed by excluding fillers that were not statistically significant. The minimal model included the *a*(あ)-group, *e*(え)-group, *o*(お)-group, *n*(ん)-group, *maa*(まあ)-group, and *sono*(その)-group fillers as fixed effects. The minimal model had a log-likelihood of −538.303 (df = 16) and an AIC of 1108.605. The likelihood ratio test revealed no statistically significant difference in fit between the full and minimal models (LR χ² = 2.96, p = 0.565 > 0.05). Furthermore, the estimates, p-values, and 95% confidence intervals for the fillers retained in the minimal model showed no notable changes. These results suggest that the estimates from the full model are generally stable and possess a certain degree of robustness.

In Table 5, the variance of the random intercept for L1 was nearly zero (Variance < 0.001, SD < 0.001), indicating that the unique effect of L1 background on proficiency is negligible. Moreover, since the VIF values for all predictor variables ranged from 1.08 to 1.26, all well below 5, multicollinearity was not considered a problem in this model.

Examination of the p-values for each filler revealed no statistically significant association for *i*(い)-group (p = 0.630 > 0.05), *u*(う)-group (p = 0.356 > 0.05), *eeto*(ええと)-group (p = 0.657 > 0.05), or *ano*(あの)-group (p = 0.191 > 0.05). Thus, no significant association was found between the frequency of these fillers and proficiency.

In contrast, among the fillers that showed a statistically significant association, higher frequency of *sono*(その)-group (Estimate = 0.226, p < .001) and *maa*(まあ)-group (Estimate = 0.028, p = 0.036 < 0.05) was significantly associated with higher proficiency. Conversely, higher frequency of *a*(あ)-group (Estimate = −0.033, p < .001), *e*(え)-group (Estimate = −0.028, p = 0.002 < 0.05), *o*(お)-group (Estimate = −0.147, p = 0.002 < 0.05), and *n*(ん)-group (Estimate = −0.049, p < .001) was significantly associated with lower proficiency. Furthermore, the 95% confidence intervals for all these fillers did not cross zero, confirming that the direction of these associations is statistically stable.

## 6. Discussion of the CLMM results

The results in Table 5 reveal that the relationship with proficiency differs depending on the type of filler. Of particular importance for addressing the research question is the finding that higher frequency of the *sono*(その)-group and *maa*(まあ)-group fillers was significantly positively associated with higher proficiency. Yamauchi [4] suggests that learners who can appropriately use *maa*(まあ) correspond to the Advanced-Mid level on the OPI, and also identifies *maa*(まあ) and *sono*(その) as items to be introduced from the intermediate level onward. The results of this study are consistent with Yamauchi [4] ‘s view, at least from the perspective of frequency of use.

Furthermore, Konishi [9] reports that the use of lexical fillers increases with proficiency. The finding that the *sono*(その)-group and *maa*(まあ)-group fillers are positively associated with higher proficiency in this study partially supports Konishi [9] ‘s results. However, the *ano*(あの)-group and *eeto*(ええと)-group fillers, which are also classified as lexical fillers, showed no significant association with proficiency. This result indicates that not all lexical fillers uniformly increase with proficiency, and that the relationship with proficiency differs across individual fillers.

However, the lack of a significant association between the *ano*(あの)-group and *eeto*(ええと)-group fillers and proficiency does not imply that these fillers are unimportant in learners’ speech. Rather, these fillers are used across multiple proficiency levels, and their overall frequency alone may not sufficiently account for differences in OPI levels. The reasons why the *sono*(その)-group and *maa*(まあ)-group fillers are associated with higher proficiency while the *ano*(あの)-group and *eeto*(ええと)-group fillers show no significant association need to be examined in terms of their respective usages and functions, and their relationship with ‘Text Type,’ one of the OPI rating criteria.

In contrast, regarding vowel-type fillers, the *a*(あ)-group, *e*(え)-group, *o*(お)-group, and *n*(ん)-group fillers were significantly associated with lower proficiency, while the *i*(い)-group and *u*(う)-group fillers showed no significant association. This result partially supports Konishi [9] ‘s finding that the use of vowel-type fillers decreases as proficiency increases. These fillers may be used by learners either as Japanese fillers or as foreign-language fillers; in either case, their overuse gives the impression that the learner’s Japanese is not very fluent.

Regarding cases where these fillers are used as Japanese fillers, Yamane [6,p. 237] states that their primary function is to adjust utterance rhythm and fill pauses. It has also been pointed out that their overuse can hinder listenability [6,p. 234].

Furthermore, Konishi [9] notes that the fillers *a*(あ), *i*(い), *u*(う), *e*(え), *o*(お), and *n*(ん) may be influenced by vowels in the learner’s native language. For example, English has vowels represented as a, e, i, o, u; Korean has vowels such as ㅏ(a), ㅣ(i), ㅜ(u), ㅔ(e), ㅗ(o); and Chinese uses Pinyin romanization with a, e, i, o, u. Ishiguro [10] points out that the use of fillers influenced by such native languages is one factor that creates unnaturalness.

Example (1) is an utterance where fillers may have been influenced by the learner’s native language. In this example, the informant is talking about their daytime work, but the target fillers (shown in bold and underlined) — mainly *a*(あ)-group and *n*(ん)-group — are overused, and the speech is fragmented, giving the impression that the learner does not speak Japanese fluently. This study presents Japanese example sentences in the following format: the romanized Japanese text is provided first, with the English translation directly below it. The headings Japanese (romanized) and English translation are used only for the first example; thereafter, they are omitted.

Example (1)

Japanese (romanized):

***aa*(あー)** hiru ***i*(い)** hi hiru, ***nn*(んー)** iroiro naa-to, shigoto-ga ari-masu-kedo, ***n*(ん)**, ***nn*(んー)** watashi-wa, ***aa*(あー)**, shachoo-ni sukejuru-ni, ***a*(あ)**, tsure-te watashi-mo issho-ni idoo-suru.

English translation:

***aa*(あー)** in the after,***i*(い)**, afternoon, ***nn*(んー)** there are var— various, jobs, but ***n*(ん)**,***nn*(んー)** I, ***aa*(あー)**, I follow the boss’s schedu— schedule, ***a*(あ)**, and I, I move around together with him.

(Data ID: 0079 / OPI level: Novice-High, partially modified)

Because the learners’ native languages in this study are diverse, it cannot be determined whether the use of the above fillers in the utterance data is influenced by the learners’ L1. This is a limitation of the study. Therefore, in this study, these fillers that may be subject to L1 influence are excluded from the analysis in the Discussion Based on Filler Usages and Functions section.

The relationship between filler use and proficiency was examined using CLMM. The results showed no significant association with proficiency for the *i*(い)-group, *u*(う)-group, *ano*(あの)-group, and *eeto*(ええと)-group fillers. In contrast, the *a*(あ)-group, *e*(え)-group, *o*(お)-group, and *n*(ん)-group fillers were significantly negatively associated with higher proficiency, while the use of *maa*(まあ)-group and *sono*(その)-group fillers were significantly positively associated with higher proficiency. Since the use of *a*(あ)-group, *i*(い)-group, *u*(う)-group, *e*(え)-group, *o*(お)-group, and *n*(ん)-group fillers is influenced by the learners’ L1, the analysis based on filler usages and functions will target the *ano*(あの)-group, *eeto*(ええと)-group, *maa*(まあ)-group, and *sono*(その)-group fillers.

## 7. Discussion based on filler usages and functions

### 7.1. Discussion based on usages of *sono*

Previous research has stated that *sono*(その) has three usages. Here, the re‑presenting type of *sono*(その) is analyzed first. The re‑presenting type of *sono*(その) is defined as the usage that reintroduces a previously mentioned item or its equivalent, while the inferential type introduces new information through inference [1].

Example (2) is a typical case of the re‑presenting type. In (2), the word ‘salary(kyuuryou)’ appears immediately after the filler *sono*(その) as well as in the preceding context. The informant states that there is a discrepancy between the rent payment date and the payday, that they started working last week, and that they will receive their salary in one week. The utterance can be divided into three sentences, but using the re‑presenting type of *sono*(その) requires at least two components: a statement about a certain matter in the preceding context, and a statement about the same matter in the following context.

Example (2)

Yachin harau no hi to kyuuryou morau no hi ga chotto chigatte, senshuu kara chanto, baito o mitsukatte hataraiterun desu kedo, ***sonoo*(そのー)**,kyuuryou ga isshuukan go ni morau koto ni narimashita.

The day I pay rent and the day I get my salary are a bit different. I found a part‑time job and have been working since last week, and ***sonoo*(そのー)** I will receive my salary in one week.

(Data ID: 0001 / OPI level: Advanced‑Mid, partially modified)

Example (3) illustrates the inferential type of *sono*(その). In (3), the informant describes their experience of contact with Japanese people. The filler *sono*(その) is used when, based on the preceding explanation, the conclusion is reached that the way to become close friends differs between Japanese and Korean people. Using the inferential type of *sono*(その) requires two components of utterance content: ‘information serving as the basis for inference’ in the preceding context (in (3), the experience of contact with Japanese people) and ‘information obtained through inference’ in the following context (in (3), the fact that the way to become close friends differs between Japanese and Korean people).

Example (3)

taihen na koto mo issho ni yatte de ichinen gurai tatta ato, hontoni nakaii shinyuu da naa to watashi dake ga omotteta mitai de, ‘ichinen wa mijikai yo ne’ tte iu fuu ni iwareta toki wa bikkuri shimashita. desu kara moshikasuru to, ***sono*(その)** naka yoku naru, ***sono*(その)**, yarikata to iimasu ka ***sono*(その)**, chikazuku chia shitashiku naru, ***sono*(その)** kyori da toka naiyou da toka te iu no ga, chigau no kana tte iu no wa, ano ookii koto desu.

We went through difficult times together, and after about a year, I thought we had become truly close friends — but apparently only I thought so. I was surprised when they said, ‘One year is short, isn’t it?’ So perhaps ***sono*(その)** the way to become close friends, ***sono*(その)** the method, shall I say, ***sono*(その)** the distance and ***sono*(その)** the content of becoming close — those things might be different. That is a big realization.

(Data ID: 0202 / OPI level: Superior, partially modified)

Based on the above analysis, it can be said that both the re‑presenting type and the inferential type share a common feature: the utterance develops on the basis of prior linguistic context. From this perspective, the re‑presenting and inferential types of *sono*(その) are considered to be associated with language production at the ‘paragraph’ level, defined as ‘a collection of sentences cohesively connected’ [5].

In contrast, as shown in Example (4), cases that convey a nuance such as ‘making an excuse’ and cannot be explained by the re‑presenting or inferential types are defined as the ‘indefinite type.’ In this usage, the production of subsequent content is not necessarily guaranteed, making it difficult to directly explain its relationship with ‘Text Type.’

Example (4)

(Scene where a husband’s infidelity has been discovered by his wife)

Wife: kore wa dou iu koto nano? chanto setsumei shinasai yo!
Husband: so, sore wa, ***sonoo*(そのー)**...
Wife: ‘What is this? Explain yourself properly!’
Husband: ‘S-, well, ***sonoo*(そのー)**  ...’ [1]

Because ‘paragraph’-level language production corresponds to the Advanced level on the OPI, the re‑presenting and inferential types of *sono*(その) may be characteristic of advanced learners. This point is examined below.

As far as the learner utterance data in this study are concerned, no instances of the ‘indefinite type’ of *sono*(その) were observed — the type that conveys a nuance such as ‘making an excuse’ as in Example (4). Therefore, the learners’ use of *sono*(その) in this study is considered to fall into either the re‑presenting or the inferential type. In Table 5, the use of *sono*(その) was significantly positively associated with higher proficiency (p < .001). It can be said that advanced learners use the re-presenting and inferential types of *sono*(その) more frequently than novice or intermediate learners.

To verify which usage of *sono*(その) learners use more frequently, we examine the data. Daikuhara [1] notes that some instances cannot be clearly groupified as either re‑presenting or inferential. However, the re‑presenting type has a typical feature: a referent mentioned in the preceding context reappears in the following context. For example, in Example (2), the word ‘salary(kyuuryou)’ appears immediately after *sono*(その) as well as in the preceding context. Similarly, in Example (5), the expressions important person(erai hito) and politician(seijika) in the preceding context of *sono*(その) (shown in bold and underlined) can be interpreted as synonymous with military personnel(gunjin) in the following context, given that the military(gun-no-hoo) appears earlier. In this study, cases where identical or synonymous words appear in the preceding and following contexts of *sono*(その) are groupified as the re‑presenting type.

Example (5)

sensoo-o okoshita toki-ni, sono- okosu saiji-ni ne, **sonoo(そのー)** ma gun, gun-no-hoo, sugoku koo ne, ano- era- erai hito-dattari, seijika-dattari ne, **sonoo(そのー)** maa gen’eki-no gunjin deshoo ne, wakamono-ni tai-shite, yoo-wa kuni-no tame-ni inochi-o sasage-ro to.

When starting a war, at the time of starting it, **sonoo(そのー)** well, the military, the military side, you know, important people, politicians, **sonoo(そのー)** well, active‑duty military personnel, I suppose, say to young people: in short, sacrifice your life for the country.

(Data ID: 0323 / OPI level: Superior, partially modified)

The groupification results are shown in Fig 2 (stacked bar graph). In Fig 2, cases groupified as the re‑presenting type are labeled ‘re‑presenting,’ and all others are labeled ‘other.’ The vertical axis represents the adjusted frequency of *sono*(その) per 1,000 words, and the horizontal axis represents each learner group.

**Fig 2:**
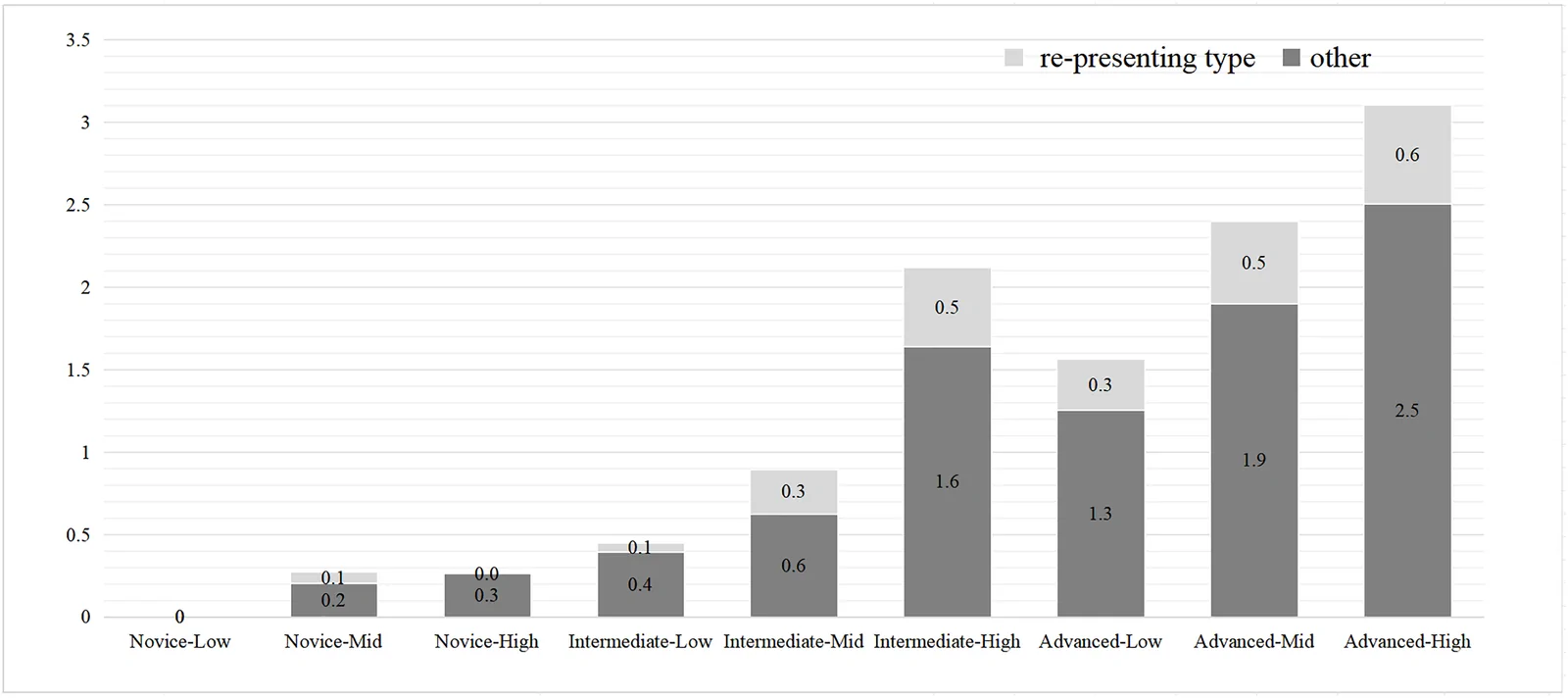
Usage of *sono*(その) (Adjusted frequency per 1,000 words).

Fig 2 confirms that typical re‑presenting type usage is relatively infrequent; in many cases, similar words do not appear in the preceding or following context of learners’ sono(その). According to Daikuhara [1], the inferential type is a symmetric counterpart to the re‑presenting type. Therefore, it is likely that most instances of *sono*(その) are used to introduce new information through inference.

The use of the filler *sono*(その) was examined above based on its usages. Because using the re‑presenting or inferential type of *sono*(その) requires at least two clauses, it became clear that these usages are associated with language production at the ‘paragraph’ level, defined in terms of ‘Text Type’ as ‘a collection of sentences cohesively connected’ [5]. Since ‘paragraph’-level language production corresponds to the Advanced level on the OPI, the re‑presenting and inferential types of *sono*(その) may be characteristic of advanced learners. To verify this point, an analysis of usage patterns was conducted. The results showed that the use of the re-presenting and inferential types was significantly positively associated with higher proficiency, while the use of the re-presenting type alone was found to be relatively infrequent.

### 7.2. Discussion based on occurrence position of *maa*

In this section, the use of *maa*(まあ) is discussed based on its occurrence position. It has usages appearing in adjacency pairs and usages appearing turn‑internally. First, an example of the adjacency‑pair usage is shown in (6) (T = tester, I = informant).

Example (6)

T: sou desu ka, anoo, 【sei B】 san wa, shumi wa nan desu ka.
I: ee, shumi, nn, ***maa*(まあ)** ongaku o kiitari, eiga o mitari.
T: I see. Um, Ms./Mr. B, what are your hobbies?
I: hobbies,um,***maa*(まあ)** listening to music or watching movies.

(Data ID: 0013 / OPI level: Advanced‑Mid, partially modified)

In (6), the informant produces *maa*(まあ) when answering the tester’s question ‘What are your hobbies?’ This can be interpreted as the informant treating the preceding question as somehow problematic (e.g., having no particular hobby but, if forced to name some, music and movies) and using *maa*(まあ) as a way to address that problem. Because the OPI is conducted in interview format, such ‘problematicity’ [12] arises primarily from the tester’s question (the first part of an adjacency pair), and the informant’s production of *maa*(まあ) appears in the response (the second part of the adjacency pair). Although the *maa*(まあ) in a response to a question is a speech act produced to answer the interlocutor’s question, it does not serve ‘functions related to text construction’ such as marking boundaries between cohesively connected sentences. Therefore, while the subsequent content after *maa*(まあ) may show sentence production, a clear association with paragraph production is not observed. Similarly, *maa*(まあ) occurring at turn‑initial position in the first part of an adjacency pair (the tester’s utterance in (6)) or in the third position turn (where the tester produces further content in response to the informant’s answer) is produced in response to the interlocutor’s speech act in the prior context, does not mark sentence boundaries, and thus no clear association with paragraph production can be confirmed.

In contrast, Example (7) illustrates the turn‑internal usage. The *maa*(まあ) in (7) appears when the informant expresses their own view on differences between Japan and Korea. As such, *maa*(まあ) occurring turn‑internally is thought to function as a marker that ‘identifies some problematicity in the speaker’s own immediately preceding utterance’ [12] and guides the subsequent utterance to address that issue. Using *maa*(まあ) turn‑internally requires the utterance to consist of a ‘presentation of problematicity’ in the preceding context and an ‘address to the problematicity’ in the following context. In this case, the clauses before and after *maa*(まあ) are cohesively connected, and *maa*(まあ) is considered to mark the boundary between them. From the perspective of ‘Text Type,’ the production of *maa*(まあ) turn‑internally is thought to be associated with paragraph‑level production.

Example (7)

yappari, denwa o kakeru hito wa, zenzen inain desu yo ne, nanka sore ga bunka, toka, densha no echiketto to iu ka, sore ga chigaimasu yo ne, de sore de, e watashi wa, ***maa*(まあ)** sore o mite, aa, nanka, nihon no densha no naka wa, nanka shizuka na kanji de kankoku yori, ii na to omoimashita.

You know, there are hardly any people who make phone calls [on trains]. That might be culture, or train etiquette — it’s different, right. So then, I, ***maa*(まあ)** seeing that, I thought, ah, somehow, the inside of Japanese trains is quiet and nicer than Korea’s.

(Data ID: 0013 / OPI level: Advanced‑Mid, partially modified)

Because ‘paragraph’-level language production corresponds to the Advanced level on the OPI, turn‑internal *maa*(まあ) may be characteristic of advanced learners. This point is examined below.

Fig 3 (100% stacked bar graph) shows the usage of *maa*(まあ) at each learner level. The vertical axis represents the percentage of *maa*(まあ) by occurrence position, the horizontal axis represents each learner group, and the data labels on the bars indicate the adjusted frequency per 1,000 words in each group. *Maa*(まあ) used at turn‑initial position in the first part, second part, or the position after the second part (third position) of an adjacency pair is abbreviated as ‘adjacency pair.’

**Fig 3:**
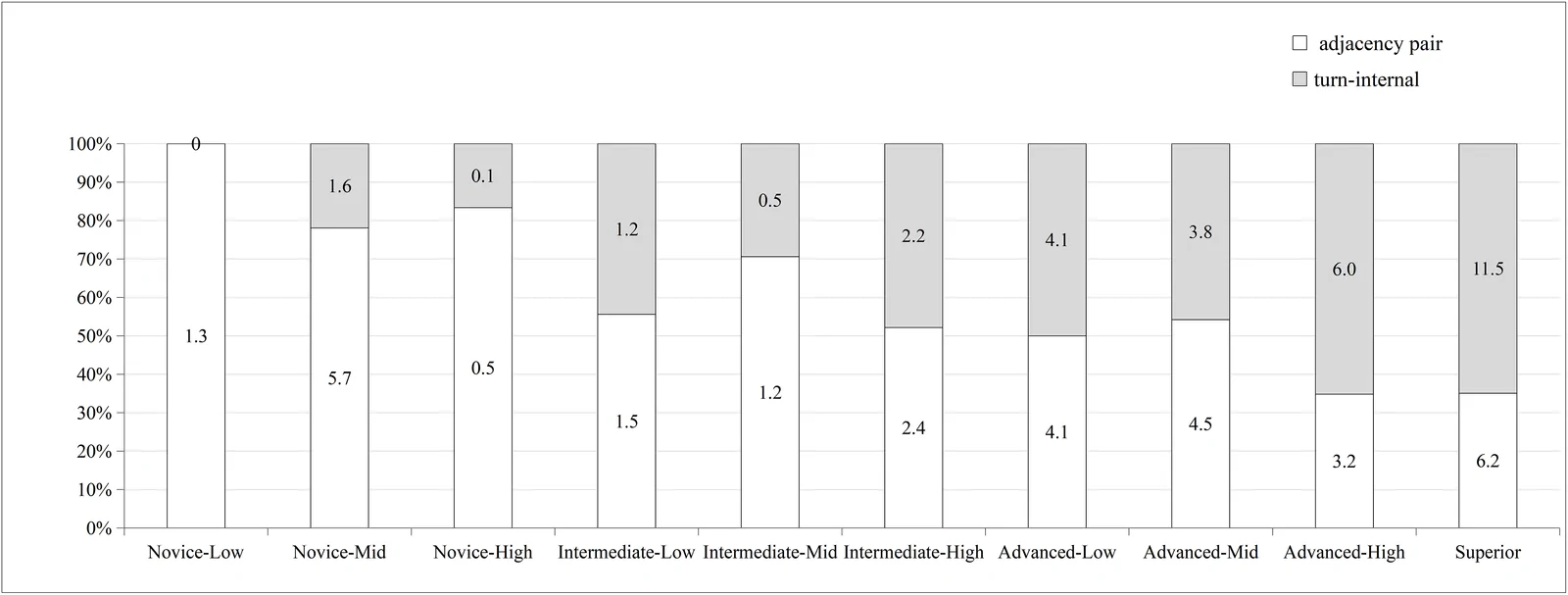
Usage of *maa*(まあ) (Adjusted frequency per 1,000 words).

According to Fig 3, in groups below the Intermediate‑Mid level (i.e., with relatively lower proficiency), the use of *maa*(まあ) in adjacency pairs is observed to a certain extent, and its proportion is higher than in the Advanced and Superior groups.

To examine the differences in the effect of *maa*(まあ) by occurrence position, *maa*(まあ) was classified into adjacency-pair and turn-internal types, and was entered as a predictor variable together with other fillers that showed statistically significant effects in the full model. The CLMM analysis was then conducted again under the same conditions. The results are shown in Table 6. Since no notable changes were observed in the coefficients or significance of the other fillers, Table 6 presents only the results for the two occurrence positions.

**Table 6:** Estimated Effects of *maa*(まあ) in Adjacency Pairs vs. Turn-Internal Positions.

	Estimate	Std. Error	z-value	p-value	VIF	95% CI
Adjacency Pairs	−0.062	0.028	−2.254	0.024	1.121	[-0.116, -0.008]
Turn-Internal	0.119	0.034	3.514	<0.001	2.004	[0.053,0.186]
AIC: 1100.083 log Lik.: −533.042(df = 17)						

Table 6 shows that adjacency-pair usage of *maa*(まあ) was significantly negatively associated with higher proficiency (Estimate = −0.062, p = 0.024 < 0.05), while turn-internal usage was significantly positively associated with higher proficiency (Estimate = 0.119, p < 0.001). A likelihood ratio test comparing the minimal model (see Table 5) and the model in Table 6 showed that the model in Table 6 had a significantly better fit than the minimal model (LR χ² = 10.52, p = 0.001; AIC = 1100.083, lower than the minimal model’s 1108.605). Thus, it can be concluded that classifying *maa*(まあ) by occurrence position and including it as a predictor variable improved model fit. For both occurrence positions, the 95% confidence intervals did not contain zero, confirming statistically significant effects. Since VIF values for all predictor variables remained below 5, no serious multicollinearity issues were present in the model in Table 6.

The use of the filler *maa*(まあ) was examined above based on occurrence position. Since turn-internal *maa*(まあ) requires two utterance components—a ‘presentation of problematicity’ in the preceding context and an ‘address to the problematicity’ in the following context—it was found to be associated with the production of ‘paragraphs’ in terms of ‘Text Type.’ Furthermore, examination of *maa*(まあ) use by occurrence position revealed that turn-internal *maa*(まあ) was significantly positively associated with higher proficiency, while adjacency-pair use was significantly negatively associated with higher proficiency. This result suggests that as proficiency increases, learners’ use of *maa*(まあ) may shift in terms of both position and function. Novice and intermediate learners tend to produce relatively short utterances, such as responses to questions, and thus *maa*(まあ) is more likely to appear at the beginning of short responses within adjacency pairs. In contrast, advanced learners are able to produce coherent multi-clausal utterances that include reasons, background information, and evaluations. In such extended discourse, they need to manage the relationship between what has been said and what follows while maintaining the flow of the ongoing utterance. Turn-internal *maa*(まあ) is used to organize discourse content and to connect preceding and following parts of the utterance. Therefore, its use is expected to increase among advanced learners who produce paragraph-level discourse.

### 7.3. Discussion of *ano* and *eeto*

A brief mention should be made of *ano*(あの) and *eeto*(ええと). As shown in Table 5, the use of these fillers showed no significant association with proficiency. First, an example of *ano*(あの) is shown in (8).

Example (8)

T: n, donna koto o shite asobimashita ka.
I: nn tatoeba, a, eiga oo, mi mimasu. ***Ano*(あの)** karaoke o, karaoke shimasu.
T: What kind of things did you do for fun?
I: Well, for example, watch movies. ***Ano*(あの)** karaoke — do karaoke.

(Data ID: 0120 / OPI level: Novice‑Mid, partially modified)

Regarding *ano*(あの), Daikuhara [1] states that it is produced when the speaker ‘has a message to utter in mind and is in the process of formulating it.’ That is, at the moment *ano*(あの) is produced in (8), the content concerning ‘karaoke’ in the following context already exists in the informant’s mind. While *ano*(あの) is thought to facilitate the construction of a ‘sentence’ or ‘word/chunks,’ it does not involve ‘reference to a meaning structure introduced through prior utterance or inference’ [1] as *sono*(その) does. Therefore, *ano*(あの) is considered less influential than *sono*(その) in terms of ‘Text Type’ and cannot be associated with paragraph‑level production.

Next, an example of *eeto*(ええと) is shown in (9).

Example (9)

T: suki na, sono terebi no bangumi tte donna n desu ka, nihon de.
I: nn maa ima made, nn, anime.
T: anime.
I: hai.
T: tatoeba.
I: ***Eeto*(ええと)**, ee, ‘Doraemon’ toka.
T: What kind of TV programs do you like, in Japan?
I: Well, up to now, nn, anime.
T: Anime.
I: Yes.
T: For example?
I: ***Eeto*(ええと)**, ee, ‘Doraemon’ or something.

(Data ID: 0383 / OPI level: Intermediate‑Mid, partially modified)

In (9), the informant produces *eeto*(ええと) while thinking of a favorite anime. *Eeto*(ええと) can be interpreted as ‘linking to a discourse management action of switching to a mental activity such as calculation’ [11]. Moreover, as noted in previous research, because *eeto*(ええと) is used when the information or linguistic form has not yet been formulated at the moment of utterance, it is difficult to directly explain its relationship with ‘Text Type.’

Thus, the fillers *ano*(あの) and *eeto*(ええと), which do not contribute to predicting proficiency, have been examined. *Ano*(あの) is considered to be associated with the production of ‘word/chunks’ in terms of ‘Text Type,’ because the linguistic form already exists in the speaker’s mind at the time of utterance. However, since it does not involve ‘reference to a meaning structure introduced through prior utterance or inference’ [1] as *sono*(その) does, an association with paragraph‑level production cannot be confirmed. Regarding *eeto*(ええと), because the linguistic form has not yet been formulated at the moment of utterance, its relationship with ‘Text Type’ is difficult to explain.

## 8. Conclusion

This study analyzed the relationship between filler use and Japanese proficiency using CLMM. The results showed no significant association with proficiency for the *i*(い)-group, *u*(う)-group, *ano*(あの)-group, and *eeto*(ええと)-group fillers. In contrast, the *a*(あ)-group, *e*(え)-group, *o*(お)-group, and *n*(ん)-group fillers were significantly negatively associated with higher proficiency, while the *maa*(まあ)-group and *sono*(その)-group fillers were significantly positively associated with higher proficiency. Furthermore, analysis of *maa*(まあ)-group and *sono*(その)-group fillers based on usage and function revealed that turn-internal *maa*(まあ) and the inferential/re‑presenting types of *sono*(その) require at least two clauses, thus associating them with ‘paragraph’-level language production in terms of ‘Text Type,’ one of the OPI rating criteria. It was also found that the use of these fillers with such functions was significantly positively associated with higher proficiency. In contrast, for *ano*(あの) and *eeto*(ええと), as well as adjacency-pair *maa*(まあ), which showed no significant association with higher proficiency, no association with paragraph-level language production was confirmed.

Based on the above analysis, this study concludes as follows: Turn‑internal *maa*(まあ) and the inferential/re‑presenting types of *sono*(その) have the function of connecting multiple clauses, and the use of these fillers associated with paragraph production can be considered one of the key linguistic factors that can help explain the relationship between filler use and the development of oral language proficiency.

This study also provides insights for filler instruction. For example, since adjacency‑pair *maa*(まあ) is observed to some extent in lower‑proficiency learner groups and at a higher proportion than in higher‑proficiency groups, it may be desirable to introduce *maa*(まあ) starting from its adjacency‑pair usage. Regarding *sono*(その), typical re‑presenting type usage is relatively infrequent, and it is likely used primarily to introduce new information through inference. Therefore, instruction should emphasize the inferential type. According to the OPI rating criteria presented in the Oral Proficiency and Its Assessment section (Table 1), advanced learners can handle unexpected situations and negotiations, while intermediate learners can handle routine situations and basic transactions. In teaching fillers, it is therefore advisable to set specific situations according to the learners’ proficiency levels. For instance, for the inferential type of *sono*(その), which introduces new information or reasons, scenarios that require providing reasons could be designed, such as explaining to club members why a training camp was canceled, or explaining to a teacher why a report submission is delayed. For adjacency-pair *maa*(まあ), on the other hand, it would be desirable to create discourse environments where such fillers are likely to occur, such as exchanging opinions about learners’ hobbies or everyday events. Furthermore, explaining the usages and conversational functions of each filler within such situations could be an effective instructional method to facilitate learners’ understanding.

A limitation of this study is that specific teaching methods for turn‑internal *maa*(まあ) and the inferential/re‑presenting types of *sono*(その) remain insufficiently clarified. Future research should aim to identify collocations and elucidate the actual use of these fillers according to their respective usages and functions, thereby contributing to concrete instructional approaches.

## Supporting information

S1 Data Learner background information. This file contains information on the native languages (L1s) and OPI proficiency levels of the 339 learners included in this study.
(XLSX)

S2 Data Analytic dataset for CLMM analysis (Tables 4 and 5). This file contains the underlying individual-level data used for the cumulative link mixed model (CLMM) analysis reported in Tables 4 and 5.
(XLSX)

S3 Data The file summarizes the number of times each filler was used by each learner, as well as the total word count in the conversational data. (XLSX)

S4 Data Analytic dataset for minimal model. (XLSX)

S5 Data Analytic dataset for Table 6. (XLSX)

S6 Data Underlying data for Fig 2. (XLSX)

S7 Data Underlying data for Fig 3. (XLSX)

S1 Code R code for CLMM analysis (Tables 4 and 5). This file contains the R code used for the cumulative link mixed model (CLMM) analysis reported in this study, including data preparation, model fitting, and result extraction.
(TXT)

S2 Code This text file contains the R code used to calculate the Variance Inflation Factors (VIF) for the fixed effects in the CLMM analysis (Tables 4 and 5), along with the corresponding output. (TXT)

S3 Code R code for the minimal model (with output). This text file contains the R code used to refit the CLMM after excluding non-significant fillers, along with the corresponding output for the minimal model.
(TXT)

S4 Code R code for likelihood ratio tests (with output). (TXT)

S5 Code R code for the CLMM analysis in Table 6 (with output). This text file contains the R code used for the CLMM analysis presented in Table 6, along with the corresponding output.
(TXT)

S6 Code R code for VIF calculation for Table 6 with output. This file contains the R code and output for the VIF calculation corresponding to the model reported in Table 6.
(TXT)

S7 Code R code for likelihood ratio test comparing the Table 6 model and the minimal model (with output). (TXT)
